# Evaluation of Fasting State-/Oral Glucose Tolerance Test-Derived Measures of Insulin Release for the Detection of Genetically Impaired β-Cell Function

**DOI:** 10.1371/journal.pone.0014194

**Published:** 2010-12-02

**Authors:** Silke A. Herzberg-Schäfer, Harald Staiger, Martin Heni, Caroline Ketterer, Martina Guthoff, Konstantinos Kantartzis, Fausto Machicao, Norbert Stefan, Hans-Ulrich Häring, Andreas Fritsche

**Affiliations:** 1 Division of Endocrinology, Diabetology, Angiology, Nephrology, and Clinical Chemistry, Department of Internal Medicine, University Hospital Tübingen and Eberhard Karls University Tübingen, Member of the German Center for Diabetes Research (DZD), Tübingen, Germany; 2 Division of Nutritional and Preventive Medicine, Department of Internal Medicine, University Hospital Tübingen, Tübingen, Germany; University of Bremen, Germany

## Abstract

**Background:**

To date, fasting state- and different oral glucose tolerance test (OGTT)-derived measures are used to estimate insulin release with reasonable effort in large human cohorts required, e.g., for genetic studies. Here, we evaluated twelve common (or recently introduced) fasting state-/OGTT-derived indices for their suitability to detect genetically determined β-cell dysfunction.

**Methodology/Principal Findings:**

A cohort of 1364 White European individuals at increased risk for type 2 diabetes was characterized by OGTT with glucose, insulin, and C-peptide measurements and genotyped for single nucleotide polymorphisms (SNPs) known to affect glucose- and incretin-stimulated insulin secretion. One fasting state- and eleven OGTT-derived indices were calculated and statistically evaluated. After adjustment for confounding variables, all tested SNPs were significantly associated with at least two insulin secretion measures (p≤0.05). The indices were ranked according to their associations' statistical power, and the ranks an index obtained for its associations with all the tested SNPs (or a subset) were summed up resulting in a final ranking. This approach revealed area under the curve (AUC)_Insulin(0-30)_/AUC_Glucose(0-30)_ as the best-ranked index to detect SNP-dependent differences in insulin release. Moreover, AUC_Insulin(0-30)_/AUC_Glucose(0-30)_, corrected insulin response (CIR), AUC_C-Peptide(0-30)_/AUC_Glucose(0-30)_, AUC_C-Peptide(0-120)_/AUC_Glucose(0-120)_, two different formulas for the incremental insulin response from 0–30 min, i.e., the insulinogenic indices (IGI)_2_ and IGI_1_, and insulin 30 min were significantly higher-ranked than homeostasis model assessment of β-cell function (HOMA-B; p<0.05). AUC_C-Peptide(0-120)_/AUC_Glucose(0-120)_ was best-ranked for the detection of SNPs involved in incretin-stimulated insulin secretion. In all analyses, HOMA-β displayed the highest rank sums and, thus, scored last.

**Conclusions/Significance:**

With AUC_Insulin(0-30)_/AUC_Glucose(0-30),_ CIR, AUC_C-Peptide(0-30)_/AUC_Glucose(0-30)_, AUC_C-Peptide(0-120)_/AUC_Glucose(0-120)_, IGI_2_, IGI_1_, and insulin 30 min, dynamic measures of insulin secretion based on early insulin and C-peptide responses to oral glucose represent measures which are more appropriate to assess genetically determined β-cell dysfunction than fasting measures, i.e., HOMA-B. Genes predominantly influencing the incretin axis may possibly be best detected by AUC_C-Peptide(0-120)_/AUC_Glucose(0-120)_.

## Introduction

Recently, genome-wide association (GWA) scans in tens of thousands of human cases and controls using high-density single nucleotide polymorphism (SNP) arrays and subsequent meta-analyses of these data provided important new insights into the genetic architecture of complex diseases [1]. In the course of these studies, a series of nearly 20 novel type 2 diabetes risk loci were identified. In smaller but extensively and thoroughly phenotyped cohorts, many of the diabetogenic alleles were shown to affect β-cell function [2]. Despite this recent scientific progress, a shortcoming of the genetic findings up to now is that the sum of all reported common GWA-derived risk alleles only marginally improves the prediction of future type 2 diabetes, when combined with established clinical parameters, and only explains about 6% of the heritability of the disease [3]. Thus, it is anticipated that many more loci remain to be discovered that act in an additive or even synergistic manner to increase the type 2 diabetes risk. Amongst others, the following strategies are currently discussed to find them: (i) use of SNP arrays of higher density, (ii) assessment of rare variants, and (iii) realization of GWA analyses using quantitative traits known to be crucially involved in the pathogenesis of type 2 diabetes, such as insulin secretion and insulin sensitivity [2]; [3].

One possibility to identify more loci affecting β-cell function is to determine insulin release in cohorts large enough to allow reliable genetic analyses. To estimate insulin release in such cohorts with reasonable effort, i.e., at low expenses in time and costs, fasting state- and several different oral glucose tolerance test (OGTT)-derived indices calculated from plasma insulin, C-peptide, and glucose concentrations are available [4]–[10]. However, which of these indices are best-suited to detect genetically determined β-cell dysfunction is unknown. Therefore, we evaluated, in this study, fasting state- (homeostasis model assessment of β-cell function [HOMA-B]) and OGTT-derived indices (insulin and C-peptide concentrations at 30 min of OGTT, insulinogenic indices [IGIs], area under the curve [AUC]_Insulin(0-30)_/AUC_Glucose(0-30)_, AUC_C-Peptide(0-30)_/AUC_Glucose(0-30)_, AUC_Insulin(0-120)_/AUC_Glucose(0-120)_, AUC_C-Peptide(0-120)_/AUC_Glucose(0-120)_, oral disposition index [DI oral], corrected insulin response [CIR], and first-phase insulin secretion) for their suitability to detect altered insulin release due to confirmed type 2 diabetes risk SNPs convincingly described to affect specific aspects of β-cell function, such as glucose-stimulated insulin secretion (GSIS), incretin-stimulated insulin secretion (ISIS), or incretin release. For this investigation, we included the type 2 diabetes risk loci/SNPs *MTNR1B* rs10830963, *HHEX* rs7923837, *CDKAL1* rs7754840, *TCF7L2* rs7903146, *WFS1* rs10010131, and *KCNQ1* rs151290.

## Materials and Methods

### Ethics statement

From all participants, informed written consent to the study was obtained, and the Ethics Committee of the Medical Faculty of the University of Tübingen approved the study protocol.

### Subjects

A cohort of 1364 White individuals was recruited from the ongoing Tübingen family study for type 2 diabetes (TÜF) that currently encompasses ∼2000 participants at increased risk for type 2 diabetes (non-diabetic individuals from Southern Germany with family history of type 2 diabetes or diagnosis of impaired fasting glycaemia) [11]. More than 99.5% of the TÜF participants are of European ancestry. All participants underwent the standard procedures of the study protocol including medical history and physical examination, assessment of smoking status and alcohol consumption habits, routine blood tests, and an OGTT. Selection of the present study cohort was based on the absence of newly diagnosed diabetes and the availability of complete sets of clinical and genotype data. Moreover, the participants were not taking any medication known to affect glucose tolerance or insulin secretion. The subject characteristics are given in Table 1. From this cohort, a subset of 274 individuals additionally underwent a frequently sampled intravenous glucose tolerance test (IVGTT).

**Table 1:** Subject characteristics including the fasting state- and OGTT-derived indices of insulin release (N = 1364).

	Count or Mean ±SD		
Parameter	NGT	IFG	IGT
Women/men (N)	661/331	83/54	95/33
Age (yrs)	37±12	43±13	41±13
BMI (kg/m^2^)	27.2±6.9	31.7±10.2	30.1±7.3
Fasting glucose (mmol/l)	4.90±0.39	5.84±0.25	5.10±0.30
Glucose 120 min (mmol/l)	5.61±1.13	6.22±0.99	8.71±0.76
Fasting insulin (pmol/l)	53.3±43.1	80.5±73.0	72.7±52.7
Fasting C-peptide (pmol/l)	578±252	773±389	673±296
HOMA-β (U/mol)*	134±115	116±106	153±104
Insulin 30 min (pmol/l)*	465±375	507±369	530±397
C-Peptide 30 min (pmol/l)*	1992±841	2182±1008	2040±910
IGI_1_ (×10^−9^)*	164±229	148±131	123±93
IGI_2_ (×10^−9^)*	51.5±41.6	46.7±36.0	50.5±38.4
DI oral (mmol^−1^)*	3.67±5.86	2.19±1.88	1.90±1.19
CIR (l×mmol^−1^×10^−9^)*	1642±1450	1141±943	1204±866
First-phase insulin secretion (pmol/l)*	1234±784	1247±893	1280±865
AUC_Insulin(0-30)_/AUC_Glucose(0-30)_ (×10^−9^)*	40.0±30.3	39.3±28.6	42.5±29.8
AUC_Insulin(0-120)_/AUC_Glucose(0-120)_ (×10^−9^)*	58.6±42.4	63.9±41.3	66.9±50.1
AUC_C-Peptide(0-30)_/AUC_Glucose_ (×10^−9^)*	201±77	198±85	192±75
AUC_C-Peptide(0-120)_/AUC_Glucose(0-120)_ (×10^−9^)*	324±105	322±116	289±100

* Seventeen subjects with calculated negative values in one or more of the twelve insulin secretion indices tested were excluded (N = 1347). AUC – area under the curve; BMI – body mass index; CIR – cleared insulin response; DI – disposition index; HOMA-B – homeostasis model assessment of beta-cell function; IFG – impaired fasting glycaemia; IGI – insulinogenic index; IGT – impaired glucose tolerance; NGT – normal glucose tolerance; OGTT – oral glucose tolerance test.

### OGTT and IVGTT

A standard 75-g OGTT was performed after a ten-hour overnight fast, and venous blood samples were drawn at time-points zero, 30, 60, 90, and 120 min for the determination of plasma glucose, insulin, and C-peptide concentrations. In those individuals who agreed to undergo the IVGTT, baseline samples (-10, -5, and 0 min) were collected before a glucose dose of 0.3 g/kg body weight was given. Blood samples for the measurement of plasma glucose and insulin were obtained at 2, 4, 6, 8, 10, 20, 30, 40, 50, and 60 min.

### Laboratory measurements

Plasma glucose was determined using a bedside glucose analyzer (glucose oxidase method, Yellow Springs Instruments, Yellow Springs, OH, USA). Plasma insulin and C-peptide concentrations were measured by commercial chemiluminescence assays for ADVIA Centaur (Siemens Medical Solutions, Fernwald, Germany) according to the manufacturer's instructions.

### Selection of loci/SNPs

From each confirmed type 2 diabetes risk locus previously reported to affect specific aspects of β-cell function, we selected one representative SNP based on the availability of genotype data and on the robustness of the SNP's β-cell effect in our cohort. As loci/SNPs associated with GSIS, we selected *MTNR1B* rs10830963 [12]; [13], *HHEX* rs7923837 [14]; [15], and *CDKAL1* rs7754840 [16]; [17]. As loci/SNPs predominantly associated with ISIS/incretin release, we selected *TCF7L2* rs7903146 [18]; [19], *WFS1* rs10010131 [20], and *KCNQ1* rs151290 [21]. All SNPs were genotyped in the whole cohort in the course of earlier studies [12]; [15]; [17]; [18]; [20]; [21] using TaqMan assays (Applied Biosystems, Foster City, CA, USA) and passed the quality controls. Details on this as well as on minor allele frequencies, genotyping success rates, and Hardy-Weinberg equilibrium are reported in the aforementioned references.

### Calculations

Insulin secretion derived from the fasting state was calculated as HOMA-B: 20·I_0_/(G_0_–3.5) with I_0_ = fasting insulin in µU/ml and G_0_ = fasting glucose in mmol/l [5]. All other insulin secretion indices were derived from the OGTT with insulin and C-peptide concentrations given in pmol/l, and glucose concentration given in mmol/l. AUCs of insulin, C-peptide, and glucose concentrations during the entire 120 min of the OGTT were calculated according to the trapezoid method as: 0.5·(0.5·c_0_+c_30_+c_60_+c_90_+0.5·c_120_) with c = concentration. AUC_Insulin(0-30)_/AUC_Glucose(0-30)_ was calculated as: (I_0_+I_30_)/(G_0_+G_30_) [9]. AUC_C-Peptide(0-30)_/AUC_Glucose(0-30)_ was calculated analogously. IGI_1_ was calculated as: (I_30_–I_0_)/(G_30_–G_0_) [10]. IGI_2_ was calculated as: (I_30_–I_0_)/G_30_ [6]. DI oral was calculated as: IGI_1_/I_0_ [8]. CIR was calculated as: 100·I_30_/[G_30_·(G_30_–3.89)] [4]. First-phase insulin secretion was calculated as: 1283+1.829·I_30_–138.7·G_30_+3.772·I_0_ [7]. Insulin sensitivity derived from the OGTT was estimated as proposed by Matsuda and DeFronzo [22]: 10000/(G_0_·I_0_·G_mean_·I_mean_)^½^. Fasting insulin clearance was calculated as CP_0_/I_0_ with CP_0_ = fasting C-peptide, insulin clearance during the OGTT was calculated as AUC_C-Peptide(0-120)_/AUC_Insulin(0-120)_. Acute insulin response (AIR) derived from the IVGTT was used as gold standard for the assessment of insulin secretion and calculated as: 0.5·(0.5·I_0_+I_2_+I_4_+I_6_+I_8_+0.5·I_10_).

### Statistical analyses

Prior to analysis, all continuous data were log*_e_*-transformed in order to approximate normal distribution. Multiple linear regression analysis was performed using the least-squares method. In the regression models, the insulin secretion parameter was chosen as dependent variable, the SNP genotype (additive inheritance model) as independent variable, and gender, age, BMI, and OGTT-derived insulin sensitivity as confounding variables. In addition, the SNP genotype (additive inheritance model) was tested as dependent variable and the insulin secretion parameter as independent variable with inclusion of the aforementioned confounders in the models. Since the critical confounding variables age, BMI, and OGTT-derived insulin sensitivity did not achieve normal distribution even after applying the ladder of powers (probably due to the inclusion/exclusion criteria of our study), we additionally performed linear regression models including these parameters as nominal variables after stratification into quartiles. A p-value ≤0.05 was considered statistically significant. Multiple linear regression analyses, *post hoc* power calculations [statistical power (1-β) and least significant number (lsn; i.e., the sample size expected to be needed to achieve statistical significance) to detect the effect size given by the default settings (square root of the sum of squares for the hypothesis divided by N)], and Wilcoxon rank sum tests were performed using the statistical software package JMP 4.0 (SAS Institute, Cary, NC, USA).

## Results

Seventeen subjects with calculated negative values in single insulin secretion measures were excluded from all analyses resulting in a final cohort of 1347 individuals. Since the phenotype (insulin release) is determined by the genotype, we started our analyses using the insulin secretion index as dependent variable and the SNP genotype (additive inheritance model) as independent variable. As expected, all tested loci/SNPs were significantly associated with at least two of the indices after adjustment for the confounding variables gender, age, BMI, and OGTT-derived insulin sensitivity (p≤0.05, Table 2; additional statistical data given in Table S1). Most secretion indices identified three, four, or five of the six tested loci/SNPs to be significantly associated with insulin release, whereas HOMA-B detected *MTNR1B* rs10830963 only. Inclusion of the confounding parameters age, BMI, and OGTT-derived insulin sensitivity as nominal variables (after stratification into quartiles) in the linear regression models resulted in very similar statistical data (Table S2). After adjustment for gender, age, and BMI, none of the SNPs showed significant association with insulin clearance either in the fasting state (p>0.1) or during the OGTT (p≥0.06).

**Table 2:** Statistical data of the SNPs' associations with indices of insulin release and ranking of the indices according to their lsn.

	*MTNR1B* rs10830963			*HHEX* rs7923837			*CDKAL1* rs7754840			*TCF7L2* rs7903146			*WFS1* rs10010131			*KCNQ1* rs151290		
Parameter	p	1-β	lsn (rank)	p	1-β	lsn (rank)	p	1-β	lsn (rank)	p	1-β	lsn (rank)	p	1-β	lsn (rank)	p	1-β	lsn (rank)
HOMA-β	<0.0001	0.99	345 (10)	0.4	0.22	4222 (12)	0.9	0.07	34337 (12)	0.3	0.30	2938 (7)	0.3	0.30	2923 (11)	0.9	0.06	42551 (12)
Insulin 30 min	<0.0001	1.00	196 (5)	0.0010	0.92	587 (11)	0.0056	0.83	779 (1)	0.4	0.22	4127 (10)	0.2	0.38	2234 (9)	0.0088	0.79	852 (6)
C-Peptide 30 min	<0.0001	1.00	241 (8)	0.0005	0.95	536 (9)	0.2	0.37	2289 (8)	0.4	0.20	4800 (11)	0.0312	0.65	1164 (2)	0.0022	0.89	657 (1)
IGI_1_	<0.0001	1.00	201 (6)	0.0002	0.96	483 (6)	0.1	0.43	1914 (7)	0.2	0.36	2376 (4)	0.07	0.52	1537 (4)	0.0212	0.70	1046 (9)
IGI_2_	<0.0001	1.00	164 (2)	0.0005	0.95	527 (8)	0.0110	0.77	894 (3)	0.3	0.28	3134 (8)	0.1	0.42	2001 (8)	0.0063	0.82	797 (4)
DI oral	<0.0001	1.00	288 (9)	0.0008	0.93	568 (10)	0.1	0.48	1715 (6)	0.3	0.27	3285 (9)	0.2	0.38	2250 (10)	0.0278	0.67	1127 (11)
CIR	<0.0001	1.00	164 (2)	0.0001	0.98	447 (3)	0.07	0.54	1487 (5)	0.1	0.40	2098 (3)	0.1	0.45	1824 (5)	0.0234	0.69	1075 (10)
First-phase insulin secretion	<0.0001	1.00	208 (7)	0.0002	0.97	468 (5)	0.0134	0.75	936 (4)	0.4	0.19	4951 (12)	0.1	0.42	1976 (7)	0.0164	0.73	981 (7)
AUC_Insulin(0-30)_/AUC_Glucose(0-30)_	<0.0001	1.00	153 (1)	0.0001	0.97	456 (4)	0.0069	0.81	810 (2)	0.2	0.31	2808 (6)	0.1	0.45	1855 (6)	0.0066	0.82	803 (5)
AUC_Insulin(0-120)_/AUC_Glucose(0-120)_	0.0002	0.97	478 (12)	<0.0001	0.99	356 (2)	0.4	0.21	4465 (10)	0.05	0.58	1361 (2)	0.5	0.17	5549 (12)	0.0194	0.71	1023 (8)
AUC_C-Peptide(0-30)_/AUC_Glucose(0-30)_	<0.0001	1.00	180 (4)	0.0003	0.96	500 (7)	0.3	0.29	2993 (9)	0.2	0.33	2580 (5)	0.0266	0.67	1112 (1)	0.0033	0.87	706 (3)
AUC_C-Peptide(0-120)_/AUC_Glucose(0-120)_	0.0001	0.97	453 (11)	<0.0001	1.00	338 (1)	0.6	0.13	8127 (11)	0.0262	0.67	1109 (1)	0.0423	0.61	1276 (3)	0.0026	0.88	677 (2)

Seventeen subjects with calculated negative values were excluded (N = 1347). Prior to multiple linear regression analysis, all continuous variables were log*_e_*-transformed to approximate normal distribution. In the multiple linear regression models, the insulin secretion parameter was chosen as dependent variable, the SNP genotype (additive inheritance model) as independent variable and gender, age, BMI, and OGTT-derived insulin sensitivity as confounding variables. AUC – area under the curve; BMI – body mass index; CIR – cleared insulin response; DI – disposition index; HOMA-B – homeostasis model assessment of beta-cell function; IGI – insulinogenic index; lsn – least significant number (sample size expected to be needed to achieve statistical significance); SNP – single nucleotide polymorphism.

To evaluate which indices are most appropriate to detect genetically determined differences in insulin release, we first calculated the *post hoc* least significant numbers for all associations and converted them into ranks with indices that displayed the lowest least significant number being the best-ranked (Table 2). Then, we summed up the ranks of each insulin secretion index obtained for all the tested SNPs and ranked the indices according to their rank sums (Table 3). Using this approach, AUC_Insulin(0-30)_/AUC_Glucose(0-30)_ was identified as the best-ranked index (Table 3). Moreover, AUC_Insulin(0-30)_/AUC_Glucose(0-30)_, CIR, AUC_C-Peptide(0-30)_/AUC_Glucose(0-30)_, AUC_C-Peptide(0-120)_/AUC_Glucose(0-120)_, IGI_2_, IGI_1_, and insulin 30 min, but not C-peptide 30 min, first-phase insulin secretion, AUC_Insulin(0-120)_/AUC_Glucose(0-120)_, or DI oral, were significantly higher-ranked than HOMA-B (p<0.05; Table 3). To avoid over-adjustment of DI oral, a secretion parameter already normalised for a rough estimate of insulin sensitivity (i.e., fasting insulin), this parameter was also tested in the absence of additional adjustment for OGTT-derived insulin sensitivity. This analysis resulted in a somewhat higher rank sum (57) that, however, had no impact on this index' overall rank (rank 11). When summing up the ranks of the indices obtained for the three loci/SNPs affecting GSIS, i.e., *MTNR1B* rs10830963, *HHEX* rs7923837, and *CDKAL1* rs7754840, AUC_Insulin(0-30)_/AUC_Glucose(0-30)_ again turned out to be the highest-ranked index (Table 3). Notably, when summing up the ranks obtained for the three loci/SNPs predominantly affecting ISIS, i.e., *TCF7L2* rs7903146, *WFS1* rs10010131, and *KCNQ1* rs151290, AUC_C-Peptide(0-120)_/AUC_Glucose(0-120)_ was the best-ranked index (Table 3). In the GSIS and ISIS subgroups, statistical analysis of the rankings was inappropriate due to the small sample sizes. In all rankings, HOMA-β displayed the highest rank sums and, thus, represented the lowest-ranked index. Assessing the SNP genotype (additive inheritance model) as dependent variable and the insulin secretion parameter as independent variable with inclusion of the aforementioned confounders in the multiple regression models yielded very similar rankings (Tables S3 and S4).

**Table 3:** Ranking of the indices of insulin release according to their rank sums.

	Overall ranking(all SNPs tested)			Ranking for detection of GSIS(*MTNR1B*, *HHEX*, and *CDKAL1* SNPs)			Ranking for detection of ISIS(*TCF7L2*, *WFS1*, and *KCNQ1* SNPs)	
Rank	Parameter	Rank sum (from lsn)	Rank	Parameter	Rank sum (from lsn)	Rank	Parameter	Rank sum (from lsn)
1	AUC_Insulin(0-30)_/AUC_Glucose(0-30)_	24*	1	AUC_Insulin(0-30)_/AUC_Glucose(0-30)_	7	1	AUC_C-Peptide(0-120)_/AUC_Glucose(0-120)_	6
2	CIR	28*	2	CIR	10	2	AUC_C-Peptide(0-30)_/AUC_Glucose(0-30)_	9
3	AUC_C-Peptide(0-30)_/AUC_Glucose(0-30)_	29*	3	IGI_2_	13	3	C-Peptide 30 min	14
	AUC_C-Peptide(0-120)_/AUC_Glucose(0-120)_	29*	4	First-phase insulin secretion	16	4	IGI_1_	17
5	IGI_2_	33*	5	Insulin 30 min	17		AUC_Insulin(0-30)_/AUC_Glucose(0-30)_	17
6	IGI_1_	36*	6	IGI_1_	19	6	CIR	18
7	C-Peptide 30 min	39	7	AUC_C-Peptide(0-30)_/AUC_Glucose(0-30)_	20	7	IGI_2_	20
8	Insulin 30 min	42*	8	AUC_C-Peptide(0-120)_/AUC_Glucose(0-120)_	23	8	AUC_Insulin(0-120)_/AUC_Glucose(0-120)_	22
	First-phase insulin secretion	42	9	AUC_Insulin(0-120)_/AUC_Glucose(0-120)_	24	9	Insulin 30 min	25
10	AUC_Insulin(0-120)_/AUC_Glucose(0-120)_	46	10	C-Peptide 30 min	25	10	First-phase insulin secretion	26
11	DI oral	55		DI oral	25	11	DI oral	30
12	HOMA-β	64	12	HOMA-B	34		HOMA-B	30

* Significantly different from HOMA-B (p<0.05; Wilcoxon rank sum test). AUC – area under the curve; CIR – cleared insulin response; DI – disposition index; GSIS – glucose-stimulated insulin secretion; HOMA-B – homeostasis model assessment of beta-cell function; IGI – insulinogenic index; ISIS – incretin-stimulated insulin secretion/incretin production; lsn – least significant number; SNP – single nucleotide polymorphism.

Interestingly, the indices that performed best in all these analyses, i.e., AUC_Insulin(0-30)_/AUC_Glucose(0-30)_ and CIR, also revealed the best correlations with IVGTT-derived AIR (both r = 0.76), and HOMA-B, the lowest ranked index, showed the weakest correlation with AIR (r = 0.64, N = 274; Table 4).

**Table 4:** Association of the fasting- and OGTT-derived indices of insulin release with IVGTT-derived AIR (N = 274).

	AIR	
Parameter	r	p
AUC_Insulin(0-30)_/AUC_Glucose(0-30)_	0.76	<0.0001
CIR	0.76	<0.0001
IGI_2_	0.75	<0.0001
First-phase insulin secretion	0.74	<0.0001
IGI_1_	0.72	<0.0001
AUC_C-Peptide(0-30)_/AUC_Glucose(0-30)_	0.72	<0.0001
Insulin 30 min	0.71	<0.0001
DI oral	0.70	<0.0001
AUC_Insulin(0-120)_/AUC_Glucose(0-120)_	0.70	<0.0001
C-Peptide 30 min	0.68	<0.0001
AUC_C-Peptide(0-120)_/AUC_Glucose(0-120)_	0.67	<0.0001
HOMA-β	0.64	<0.0001

Prior to multiple linear regression analysis, all continuous variables were log*_e_*-transformed to approximate normal distribution. In the multiple linear regression models, AIR was chosen as dependent variable, the fasting-/OGTT-derived insulin secretion index as independent variable and gender, age, BMI, and OGTT-derived insulin sensitivity as confounding variables. AIR – acute insulin response; AUC – area under the curve; CIR – cleared insulin response; DI – disposition index; HOMA-B – homeostasis model assessment of beta-cell function; IGI – insulinogenic index; IVGTT – intravenous glucose tolerance test; OGTT – oral glucose tolerance test.

## Discussion

In this study, we intended to identify, among twelve fasting state- and common (or recently introduced) OGTT-derived measures feasible for genetic studies in large cohorts, the indices best-suited to detect genetically determined alterations of insulin release. Since the suitability of the indices for detection of altered β-cell function may depend on the SNPs' pathomechanisms, we additionally analysed the SNPs affecting GSIS separately from those affecting the incretin axis (ISIS or incretin release). It was not the primary aim of this study to evaluate the performance of the fasting state- and OGTT-derived estimates of insulin secretion by comparing them with gold standard measures derived from laborious and expensive methods, such as IVGTT or hyperglycemic clamp.

Using summation of the ranks derived from *post hoc* least significant numbers, we show here that AUC_Insulin(0-30)_/AUC_Glucose(0-30)_, a recently proposed index validated against first-phase insulin release in a frequently sampled IVGTT [9], represents the best-ranked index for the detection of SNP effects on overall insulin release as well as on GSIS. By contrast, loci/SNPs affecting the incretin axis may be better captured by AUC_C-Peptide(0-120)_/AUC_Glucose(0-120)_. One explanation for this divergent result is that plasma concentrations of incretins, as compared to plasma glucose, do not rapidly decline after having reached their maximum during the first 60 min of the OGTT, but remain elevated until the end of the protocol [18]; [21]. Thus, OGTT-induced levels of incretins, compared to glucose, may exert more prolonged effects on the β-cell which are best assessed using indices covering the entire OGTT period. The observation that AUC_Insulin(0-120)_/AUC_Glucose(0-120)_ is not among the best-suited indices to detect alterations of the incretin axis may be due to the shorter circulating half-life of insulin as compared to C-peptide [23].

AUC_Insulin(0-30)_/AUC_Glucose(0-30)_, CIR, AUC_C-Peptide(0-30)_/AUC_Glucose(0-30)_, AUC_C-Peptide(0-120)_/AUC_Glucose(0-120)_, IGI_2_, IGI_1_, and insulin 30 min significantly outperformed HOMA-B in the detection of genetically determined differences in overall insulin release, and thus are clearly superior to HOMA-B in this regard. Since the ranks of C-peptide 30 min, first-phase insulin secretion, AUC_Insulin(0-120)_/AUC_Glucose(0-120)_, and DI oral were statistically indistinguishable from that of HOMA-B – and HOMA-B displayed the lowest ranks in all analyses –, these indices may not be recommended for genetic studies aimed at the identification of novel loci/SNPs affecting β-cell function. The validity of the OGTT-derived index AUC_Insulin(0-30)_/AUC_Glucose(0-30)_ as a preferable proxy for the assessment of β-cell function in large genetic studies is underscored by its strong correlation with IVGTT-derived AIR. In these latter analyses with IVGTT-derived AIR as gold standard, HOMA-B again revealed the weakest correlation and, thus, was confirmed to be less useful for the detection of impaired β-cell function.

A limitation of our study is that the results were generated in a single study population and, thus, clearly need replication in other comparably genotyped and phenotyped cohorts of similar or larger sample size. Furthermore, the ranking of the insulin secretion indices may also depend on the ethnicity. Since our study cohort was nearly exclusively comprised of White European subjects, similar analyses in other ethnicities would be interesting.

Finally, we conclude that, according to our data, AUC_Insulin(0-30)_/AUC_Glucose(0-30)_, along with CIR, AUC_C-Peptide(0-30)_/AUC_Glucose(0-30)_, AUC_C-Peptide(0-120)_/AUC_Glucose(0-120)_, IGI_2_, IGI_1_, and insulin 30 min, represents an appropriate surrogate parameter to assess genetically determined β-cell dysfunction. HOMA-B, DI oral, AUC_Insulin(0-120)_/AUC_Glucose(0-120)_, first-phase insulin secretion, and C-peptide 30 min, however, are of limited informative value for genetic studies on β-cell function in humans. The influence of genes on ISIS or incretin release may possibly be better detected by calculating AUC_C-Peptide_/AUC_Glucose_. These findings, if replicated in comparably sized and phenotyped cohorts, should facilitate the identification of novel loci/SNPs affecting insulin release in large cohorts metabolically characterized by OGTT with glucose, insulin, and C-peptide measurements.

## Supporting Information

Table S1 Additional statistical data of the SNPs' associations with indices of insulin release. Given are the estimate and the standard deviation of the minor allele's effect. Seventeen subjects with calculated negative values were excluded (N = 1347). Prior to multiple linear regression analysis, all continuous variables were loge-transformed to approximate normal distribution. In the multiple linear regression models, the insulin secretion parameter was chosen as dependent variable, the SNP genotype (additive inheritance model) as independent variable and gender, age, BMI, and OGTT-derived insulin sensitivity as confounding variables. AUC - area under the curve; BMI - body mass index; CIR - cleared insulin response; DI - disposition index; HOMA-B - homeostasis model assessment of beta-cell function; IGI - insulinogenic index; SD - standard deviation; SNP - single nucleotide polymorphism.
(0.07 MB DOC)

Table S2 Statistical data of the SNPs' associations with indices of insulin release using the covariates age, BMI, and OGTT-derived insulin sensitivity as nominal variables (stratified in quartiles). Given are the p-value, estimate and the standard deviation of the minor allele's effect. Seventeen subjects with calculated negative values were excluded (N = 1347). In the multiple linear regression models, the insulin secretion parameter was chosen as dependent variable, the SNP genotype (additive inheritance model) as independent variable and gender, age, BMI, and OGTT-derived insulin sensitivity as confounding variables. AUC - area under the curve; BMI - body mass index; CIR - cleared insulin response; DI - disposition index; HOMA-B - homeostasis model assessment of beta-cell function; IGI - insulinogenic index; SD - standard deviation; SNP - single nucleotide polymorphism.
(0.07 MB DOC)

Table S3 Statistical data of the SNPs' associations with indices of insulin release using the genotype as dependent variable. Seventeen subjects with calculated negative values were excluded (N = 1347). Prior to multiple linear regression analysis, all continuous variables were loge-transformed to approximate normal distribution. In the multiple linear regression models, the SNP genotype (additive inheritance model) was chosen as dependent variable, the insulin secretion parameter as independent variable and gender, age, BMI, and OGTT-derived insulin sensitivity as confounding variables. AUC - area under the curve; BMI - body mass index; CIR - cleared insulin response; DI - disposition index; HOMA-B - homeostasis model assessment of beta-cell function; IGI - insulinogenic index; lsn - least significant number (sample size expected to be needed to achieve statistical significance); SNP - single nucleotide polymorphism.
(0.08 MB DOC)

Table S4 Ranking of the indices of insulin release according to their rank sums derived from the statistics presented in Supplemental Table S3 (genotype as dependent variable). * Significantly different from HOMA-B (p<0.05; Wilcoxon rank sum test). AUC - area under the curve; CIR - cleared insulin response; DI - disposition index; GSIS - glucose-stimulated insulin secretion; HOMA-B - homeostasis model assessment of beta-cell function; IGI - insulinogenic index; ISIS - incretin-stimulated insulin secretion/incretin production; lsn - least significant number; SNP - single nucleotide polymorphism.
(0.06 MB DOC)
