# Supplementary material for: Evaluation of Fasting State-/Oral Glucose Tolerance Test-Derived Measures of Insulin Release for the Detection of Genetically Impaired β-Cell Function
Source: PLoS One. 2010 Dec 2;5(12):e14194. doi: 10.1371/journal.pone.0014194 (PMC2996282; doi:10.1371/journal.pone.0014194)
Supplement: Table S2 — Statistical data of the SNPs' associations with indices of insulin release using the covariates age, BMI, and OGTT-derived insulin sensitivity as nominal variables (stratified in quartiles). Given are the p-value, estimate and the standard deviation of the minor allele's effect. Seventeen subjects with calculated negative values were excluded (N = 1347). In the multiple linear regression models, the insulin secretion parameter was chosen as dependent variable, the SNP genotype (additive inheritance model) as independent variable and gender, age, BMI, and OGTT-derived insulin sensitivity as confounding variables. AUC - area under the curve; BMI - body mass index; CIR - cleared insulin response; DI - disposition index; HOMA-B - homeostasis model assessment of beta-cell function; IGI - insulinogenic index; SD - standard deviation; SNP - single nucleotide polymorphism. (0.07 MB DOC) [file pone.0014194.s002.doc]

**Table S2.** Statistical data of the SNPs’ associations with indices of insulin release using the covariates age, BMI, and OGTT-derived insulin sensitivity as nominal variables (stratified in quartiles)

|  | ***MTNR1B* rs10830963** | | | ***HHEX* rs7923837** | | | ***CDKAL1* rs7754840** | | | ***TCF7L2* rs7903146** | | | ***WFS1* rs10010131** | | | ***KCNQ1* rs151290** | | |
| --- | --- | --- | --- | --- | --- | --- | --- | --- | --- | --- | --- | --- | --- | --- | --- | --- | --- | --- |
| **Parameter** | **p** | **Est.** | **SD** | **p** | **Est.** | **SD** | **p** | **Est.** | **SD** | **p** | **Est.** | **SD** | **p** | **Est.** | **SD** | **p** | **Est.** | **SD** |
| HOMA-Β | 0.0001 | -0.0778 | 0.0182 | 0.6 | 0.0138 | 0.0178 | 1.0 | -0.0012 | 0.0180 | 0.4 | -0.0231 | 0.0188 | 0.6 | 0.0031 | 0.0172 | 0.8 | 0.0106 | 0.0206 |
| Insulin 30min | <0.0001 | -0.1077 | 0.0189 | 0.0054 | 0.0591 | 0.0186 | 0.0248 | -0.0509 | 0.0188 | 0.3 | -0.0221 | 0.0197 | 0.2 | 0.0310 | 0.0180 | 0.0120 | 0.0560 | 0.0215 |
| C-Peptide 30min | <0.0001 | -0.0744 | 0.0135 | 0.0017 | 0.0402 | 0.0133 | 0.3 | -0.0216 | 0.0134 | 0.4 | -0.0182 | 0.0141 | 0.0323 | 0.0337 | 0.0129 | 0.0026 | 0.0379 | 0.0154 |
| IGI_1_ | <0.0001 | -0.1679 | 0.0278 | 0.0009 | 0.0999 | 0.0273 | 0.2 | -0.0476 | 0.0276 | 0.2 | -0.0457 | 0.0289 | 0.09 | 0.0555 | 0.0265 | 0.0150 | 0.0690 | 0.0316 |
| IGI_2_ | <0.0001 | -0.1515 | 0.0231 | 0.0018 | 0.0804 | 0.0228 | 0.0312 | -0.0606 | 0.0230 | 0.3 | -0.0284 | 0.0241 | 0.2 | 0.0407 | 0.0221 | 0.0060 | 0.0726 | 0.0263 |
| DI oral | <0.0001 | -0.1432 | 0.0284 | 0.0015 | 0.0989 | 0.0278 | 0.1 | -0.0550 | 0.0281 | 0.4 | -0.0283 | 0.0294 | 0.2 | 0.0478 | 0.0270 | 0.0394 | 0.0637 | 0.0322 |
| CIR | <0.0001 | -0.1810 | 0.0271 | 0.0005 | 0.1008 | 0.0267 | 0.1 | -0.0538 | 0.0270 | 0.2 | -0.0442 | 0.0283 | 0.2 | 0.0460 | 0.0259 | 0.0149 | 0.0694 | 0.0309 |
| First-phase insulin secretion | <0.0001 | -0.1017 | 0.0178 | 0.0017 | 0.0602 | 0.0175 | 0.06 | -0.0417 | 0.0177 | 0.5 | -0.0194 | 0.0185 | 0.2 | 0.0274 | 0.0170 | 0.0094 | 0.0530 | 0.0203 |
| AUC_Insulin(0-30)_/ AUC_Glucose(0-30)_ | <0.0001 | -0.1160 | 0.0179 | 0.0013 | 0.0627 | 0.0176 | 0.0319 | -0.0467 | 0.0178 | 0.2 | -0.0263 | 0.0187 | 0.2 | 0.0297 | 0.0171 | 0.0072 | 0.0538 | 0.0204 |
| AUC_Insulin(0-120)_/ AUC_Glucose(0-120)_ | 0.0036 | -0.0507 | 0.0152 | 0.0003 | 0.0578 | 0.0148 | 0.6 | -0.0142 | 0.0149 | 0.05 | -0.0338 | 0.0156 | 0.5 | 0.0167 | 0.0143 | 0.0269 | 0.0441 | 0.0171 |
| AUC_C-Peptide(0-30)_/ AUC_Glucose(0-30)_ | <0.0001 | -0.0826 | 0.0128 | 0.0014 | 0.0391 | 0.0126 | 0.4 | -0.0168 | 0.0127 | 0.3 | -0.0204 | 0.0133 | 0.0289 | 0.0323 | 0.0122 | 0.0019 | 0.0299 | 0.0145 |
| AUC_C-Peptide(0-120)_/ AUC_Glucose(0-120)_ | 0.0002 | -0.0503 | 0.0121 | <0.0001 | 0.0506 | 0.0118 | 0.8 | -0.0083 | 0.0119 | 0.0238 | -0.0339 | 0.0125 | 0.0338 | 0.0277 | 0.0114 | 0.0021 | 0.0372 | 0.0136 |

Given are the p-value, estimate and the standard deviation of the minor allele’s effect. Seventeen subjects with calculated negative values were excluded (N=1347). In the multiple linear regression models, the insulin secretion parameter was chosen as dependent variable, the SNP genotype (additive inheritance model) as independent variable and gender, age, BMI, and OGTT-derived insulin sensitivity as confounding variables. AUC – area under the curve; BMI – body mass index; CIR – cleared insulin response; DI – disposition index; HOMA-B – homeostasis model assessment of beta-cell function; IGI – insulinogenic index; SD – standard deviation; SNP – single nucleotide polymorphism
