# Supplementary material for: Evaluation of Fasting State-/Oral Glucose Tolerance Test-Derived Measures of Insulin Release for the Detection of Genetically Impaired β-Cell Function
Source: PLoS One. 2010 Dec 2;5(12):e14194. doi: 10.1371/journal.pone.0014194 (PMC2996282; doi:10.1371/journal.pone.0014194)
Supplement: Table S3 — Statistical data of the SNPs' associations with indices of insulin release using the genotype as dependent variable. Seventeen subjects with calculated negative values were excluded (N = 1347). Prior to multiple linear regression analysis, all continuous variables were loge-transformed to approximate normal distribution. In the multiple linear regression models, the SNP genotype (additive inheritance model) was chosen as dependent variable, the insulin secretion parameter as independent variable and gender, age, BMI, and OGTT-derived insulin sensitivity as confounding variables. AUC - area under the curve; BMI - body mass index; CIR - cleared insulin response; DI - disposition index; HOMA-B - homeostasis model assessment of beta-cell function; IGI - insulinogenic index; lsn - least significant number (sample size expected to be needed to achieve statistical significance); SNP - single nucleotide polymorphism. (0.08 MB DOC) [file pone.0014194.s003.doc]

**Table S3.** Statistical data of the SNPs’ associations with indices of insulin release using the genotype as dependent variable

|  | ***MTNR1B* rs10830963** | | | ***HHEX* rs7923837** | | | ***CDKAL1* rs7754840** | | | ***TCF7L2* rs7903146** | | | ***WFS1* rs10010131** | | | ***KCNQ1* rs151290** | | |
| --- | --- | --- | --- | --- | --- | --- | --- | --- | --- | --- | --- | --- | --- | --- | --- | --- | --- | --- |
| **Parameter** | **p** | **1-β** | **lsn (rank)** | **p** | **1-β** | **lsn (rank)** | **p** | **1-β** | **lsn (rank)** | **p** | **1-β** | **lsn (rank)** | **p** | **1-β** | **lsn (rank)** | **p** | **1-β** | **lsn (rank)** |
| HOMA-Β | <0.0001 | 1.00 | 223 (10) | 0.2 | 0.24 | 3313 (12) | 0.8 | 0.06 | 52152 (12) | 0.1 | 0.30 | 2465 (6) | 1.0 | 0.05 | 1730572 (12) | 1.0 | 0.05 | 1600776 (12) |
| Insulin 30min | <0.0001 | 1.00 | 128 (5) | 0.0002 | 0.96 | 384 (9) | 0.0013 | 0.90 | 500 (1) | 0.5 | 0.10 | 12828 (12) | 0.09 | 0.39 | 1828 (9) | 0.0117 | 0.71 | 814 (4) |
| C-Peptide 30min | <0.0001 | 1.00 | 155 (8) | 0.0008 | 0.92 | 459 (11) | 0.07 | 0.45 | 1541 (8) | 0.3 | 0.18 | 4871 (8) | 0.0090 | 0.74 | 759 (2) | 0.0184 | 0.65 | 932 (6) |
| IGI_1_ | <0.0001 | 1.00 | 133 (6) | <0.0001 | 0.98 | 323 (6) | 0.0456 | 0.52 | 1295 (7) | 0.1 | 0.32 | 2369 (5) | 0.0424 | 0.53 | 1257 (4) | 0.0434 | 0.52 | 1268 (10) |
| IGI_2_ | <0.0001 | 1.00 | 108 (2) | <0.0001 | 0.97 | 342 (7) | 0.0027 | 0.85 | 575 (3) | 0.4 | 0.13 | 7553 (11) | 0.08 | 0.42 | 1690 (6) | 0.0083 | 0.75 | 744 (1) |
| DI oral | <0.0001 | 1.00 | 190 (9) | 0.0002 | 0.96 | 373 (8) | 0.0306 | 0.58 | 1107 (6) | 0.4 | 0.14 | 6701 (10) | 0.07 | 0.44 | 1584 (5) | 0.0322 | 0.57 | 1128 (8) |
| CIR | <0.0001 | 1.00 | 108 (2) | <0.0001 | 0.99 | 300 (3) | 0.0216 | 0.63 | 981 (5) | 0.1 | 0.33 | 2206 (4) | 0.09 | 0.40 | 1772 (7) | 0.0390 | 0.54 | 1215 (9) |
| First-phase insulin secretion | <0.0001 | 1.00 | 137 (7) | <0.0001 | 0.98 | 319 (5) | 0.0045 | 0.81 | 641 (4) | 0.3 | 0.17 | 5187 (9) | 0.1 | 0.34 | 2191 (10) | 0.0188 | 0.65 | 937 (7) |
| AUC_Insulin(0-30)_/ AUC_Glucose(0-30)_ | <0.0001 | 1.00 | 101 (1) | <0.0001 | 0.99 | 302 (4) | 0.0017 | 0.88 | 525 (2) | 0.3 | 0.19 | 4456 (7) | 0.09 | 0.40 | 1804 (8) | 0.0125 | 0.70 | 830 (5) |
| AUC_Insulin(0-120)_/ AUC_Glucose(0-120)_ | <0.0001 | 0.98 | 314 (12) | <0.0001 | 1.00 | 241 (1) | 0.2 | 0.27 | 2891 (10) | 0.06 | 0.5 | 1422 (2) | 0.3 | 0.20 | 4155 (11) | 0.0100 | 0.73 | 781 (3) |
| AUC_C-Peptide(0-30)_/ AUC_Glucose(0-30)_ | <0.0001 | 1.00 | 116 (4) | 0.0004 | 0.94 | 414 (10) | 0.1 | 0.34 | 2153 (9) | 0.1 | 0.34 | 2171 (3) | 0.0088 | 0.75 | 754 (1) | 0.07 | 0.44 | 1576 (11) |
| AUC_C-Peptide(0-120)_/ AUC_Glucose(0-120)_ | <0.0001 | 0.99 | 291 (11) | <0.0001 | 1.00 | 253 (2) | 0.4 | 0.13 | 8100 (11) | 0.0085 | 0.75 | 747 (1) | 0.0171 | 0.66 | 911 (3) | 0.0091 | 0.74 | 760 (2) |

Seventeen subjects with calculated negative values were excluded (N=1347). Prior to multiple linear regression analysis, all continuous variables were log*_e_*-transformed to approximate normal distribution. In the multiple linear regression models, the SNP genotype (additive inheritance model) was chosen as dependent variable, the insulin secretion parameter as independent variable and gender, age, BMI, and OGTT-derived insulin sensitivity as confounding variables. AUC – area under the curve; BMI – body mass index; CIR – cleared insulin response; DI – disposition index; HOMA-B – homeostasis model assessment of beta-cell function; IGI – insulinogenic index; lsn – least significant number (sample size expected to be needed to achieve statistical significance); SNP – single nucleotide polymorphism
