# Supplementary material for: Evaluation of Fasting State-/Oral Glucose Tolerance Test-Derived Measures of Insulin Release for the Detection of Genetically Impaired β-Cell Function
Source: PLoS One. 2010 Dec 2;5(12):e14194. doi: 10.1371/journal.pone.0014194 (PMC2996282; doi:10.1371/journal.pone.0014194)
Supplement: Table S4 — Ranking of the indices of insulin release according to their rank sums derived from the statistics presented in Supplemental Table S3 (genotype as dependent variable). * Significantly different from HOMA-B (p<0.05; Wilcoxon rank sum test). AUC - area under the curve; CIR - cleared insulin response; DI - disposition index; GSIS - glucose-stimulated insulin secretion; HOMA-B - homeostasis model assessment of beta-cell function; IGI - insulinogenic index; ISIS - incretin-stimulated insulin secretion/incretin production; lsn - least significant number; SNP - single nucleotide polymorphism. (0.06 MB DOC) [file pone.0014194.s004.doc]

**Table S4.** Ranking of the indices of insulin release according to their rank sums derived from the statistics presented in Supplemental Table 3 (genotype as dependent variable)

|  | **Overall ranking**  **(all SNPs tested)** | |  |  | **Ranking for detection of GSIS**  **(*MTNR1B*, *HHEX*, and *CDKAL1* SNPs)** | |  |  | **Ranking for detection of ISIS**  **(*TCF7L2*, *WFS1*, and *KCNQ1* SNPs)** | |
| --- | --- | --- | --- | --- | --- | --- | --- | --- | --- | --- |
| **Rank** | **Parameter** | **Rank sum (from lsn)** |  | **Rank** | **Parameter** | **Rank sum (from lsn)** |  | **Rank** | **Parameter** | **Rank sum (from lsn)** |
| 1 | AUC_Insulin(0-30)_/AUC_Glucose(0-30)_ | 27* |  | 1 | AUC_Insulin(0-30)_/AUC_Glucose(0-30)_ | 7 |  | 1 | AUC_C-Peptide(0-120)_/AUC_Glucose(0-120)_ | 6 |
| 2 | CIR | 30* |  | 2 | CIR | 10 |  | 2 | AUC_C-Peptide(0-30)_/AUC_Glucose(0-30)_ | 15 |
|  | AUC_C-Peptide(0-120)_/AUC_Glucose(0-120)_ | 30* |  | 3 | IGI_2_ | 12 |  | 3 | C-Peptide 30min | 16 |
|  | IGI_2_ | 30* |  | 4 | Insulin 30min | 15 |  |  | AUC_Insulin(0-120)_/AUC_Glucose(0-120)_ | 16 |
| 5 | AUC_C-Peptide(0-30)_/AUC_Glucose(0-30)_ | 38* |  | 5 | First-phase insulin secretion | 16 |  | 5 | IGI_2_ | 18 |
|  | IGI_1_ | 38* |  | 6 | IGI_1_ | 19 |  | 6 | IGI_1_ | 19 |
| 7 | AUC_Insulin(0-120)_/AUC_Glucose(0-120)_ | 39 |  | 7 | AUC_C-Peptide(0-30)_/AUC_Glucose(0-30)_ | 23 |  | 7 | AUC_Insulin(0-30)_/AUC_Glucose(0-30)_ | 20 |
| 8 | Insulin 30min | 40 |  |  | AUC_Insulin(0-120)_/AUC_Glucose(0-120)_ | 23 |  |  | CIR | 20 |
| 9 | First-phase insulin secretion | 42* |  |  | DI oral | 23 |  | 9 | DI oral | 23 |
| 10 | C-Peptide 30min | 43* |  | 10 | AUC_C-Peptide(0-120)_/AUC_Glucose(0-120)_ | 24 |  | 10 | Insulin 30min | 25 |
| 11 | DI oral | 46* |  | 11 | C-Peptide 30min | 27 |  | 11 | First-phase insulin secretion | 26 |
| 12 | HOMA-Β | 64 |  | 12 | HOMA-B | 34 |  | 12 | HOMA-B | 30 |

* Significantly different from HOMA-B (p<0.05; Wilcoxon rank sum test). AUC – area under the curve; CIR – cleared insulin response; DI – disposition index; GSIS – glucose-stimulated insulin secretion; HOMA-B – homeostasis model assessment of beta-cell function; IGI – insulinogenic index; ISIS – incretin-stimulated insulin secretion/incretin production; lsn – least significant number; SNP – single nucleotide polymorphism
